# Inspiratory laryngeal stridor as the main feature of progressive encephalomyelitis with rigidity and myoclonus: a case report and literature review

**DOI:** 10.1186/s12883-022-02555-y

**Published:** 2022-01-28

**Authors:** Qingyang Yao, Maolin Fu, Lijie Ren, Caihong Lin, Liming Cao

**Affiliations:** 1 Department of Neurology, The First Hospital of Quanzhou Affiliated to Fujian Medical University, Quanzhou, Fujian Province China; 2 Department of Neurology, the 910th Hospital of the Joint Logistics Support Force of the Chinese PLA, Quanzhou, Fujian Province China; 3 Department of Neurology, The First Affiliated Hospital of Shenzhen University, 3002 Sungang West Road, Futian District, Shenzhen, 518000 China

**Keywords:** Anti-glycine receptor antibody, Progressive encephalomyelitis with rigidity and myoclonus, Inspiratory laryngeal stridor, Epilepsy, Case report

## Abstract

**Background:**

Progressive encephalomyelitis with rigidity and myoclonus (PERM) is an acute, potentially life-threatening, yet curable neuro-immunological disease characterized by spasms, muscular rigidity, and brainstem and autonomic dysfunction. The clinical features of glycine receptor (GlyR) antibody-positive PERM may be overlooked, particularly with some unusual symptoms.

**Case presentation:**

A 52-year-old man was admitted to the hospital for evaluation of tension headache for 20 days and mild dysarthria. These symptoms were followed by panic, profuse sweating, severe dysarthria, dizziness, unsteady gait, and paroxysmal muscle spasms. Brain magnetic resonance imaging and cerebrospinal fluid analysis were normal. The patient’s condition steadily deteriorated. He repeatedly presented with rigidity, panic attacks, severe anxiety, paroxysmal inspiratory laryngeal stridor, cyanosis of the lips, and intractable epilepsy. Electromyography showed multiple myoclonic seizures, a single generalized tonic-clonic seizure, and a single generalized tonic seizure. Screening for autoimmune encephalitis antibodies revealed anti-GlyR antibodies in his cerebrospinal fluid. Immunomodulatory pulse therapy with steroids and immunoglobulin resulted in expeditious improvement of the symptoms within 2 weeks, and a follow-up at 5 weeks showed consistent clinical improvement.

**Conclusion:**

Our case highlights that inspiratory laryngeal stridor is an important symptom of PERM. Our observation widens the spectrum of the clinical presentation of anti-GlyR antibody-positive PERM, where early identification is a key to improving prognosis.

**Supplementary Information:**

The online version contains supplementary material available at 10.1186/s12883-022-02555-y.

## Background

Progressive encephalomyelitis with rigidity and myoclonus (PERM) is an acute, potentially life-threatening, yet curable neuroimmunological disease characterized by spasms, muscular rigidity, and brainstem and autonomic dysfunction. The clinical features, especially some unusual symptoms, may be overlooked in general neurologic exams. Since the initial description of glycine receptor antibodies (GlyR-Abs) in a patient with PERM in 2008, these antibodies have been reported in PERM and stiff-person syndrome (SPS) [1]. GlyR-Abs were observed primarily in adults [2] and have been reported in over 100 patients [1]. The main clinical symptoms of PERM are early brainstem involvement, including oculomotor disturbance and bulbar symptoms. Additional symptoms include spinal cord symptoms, autonomic symptoms [3], muscle spasms, cramps, and myoclonus. A relatively rare sign is epilepsy, stimulus-evoked startle, and respiratory failure. To date, repeated inspiratory laryngeal stridor as a symptom of PERM has not been reported. We present an illustrative case report and discuss relevant literature.

## Case description

**Fig. 1:**
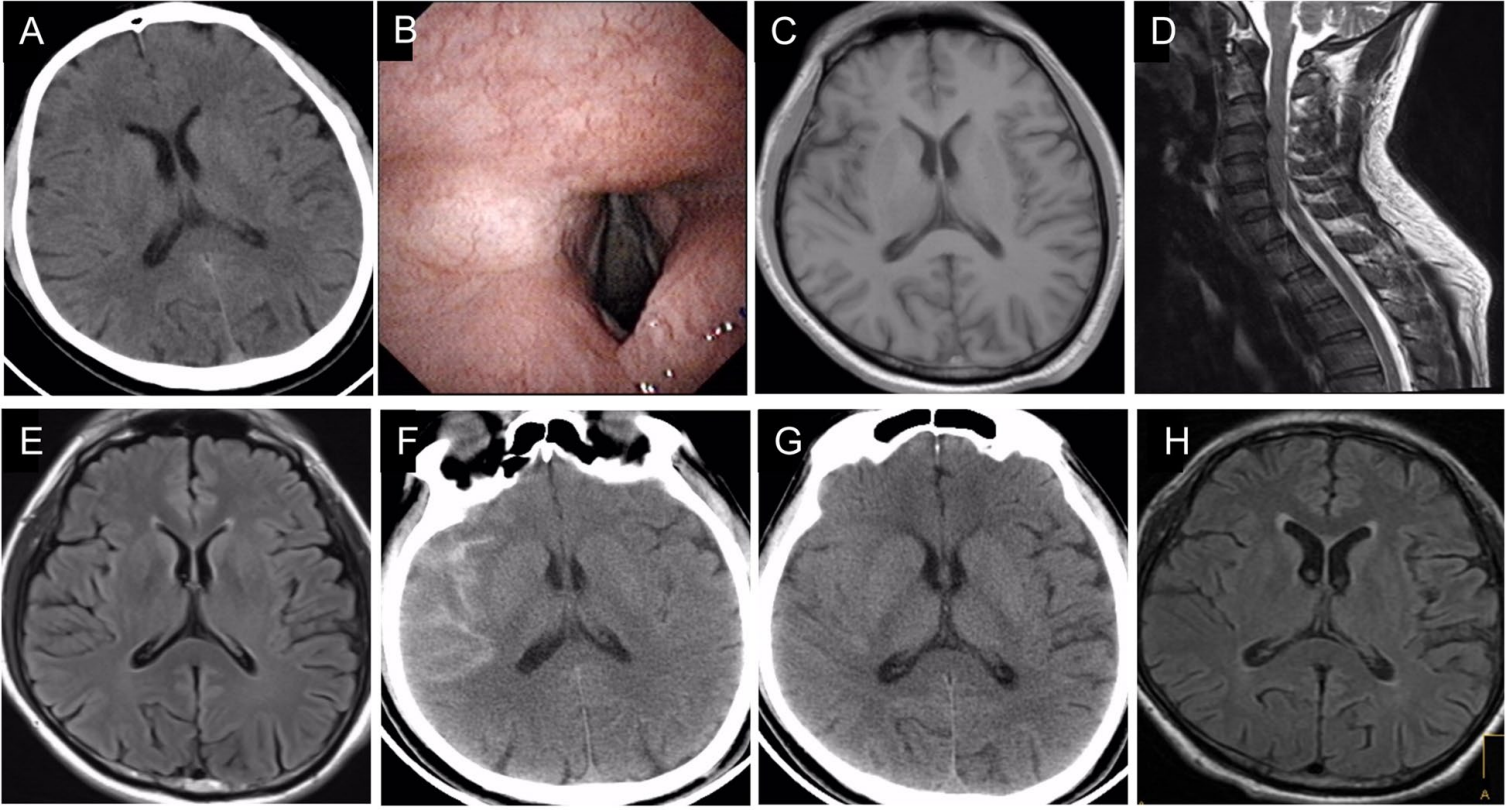
Brain computed tomography (CT) showed no obvious abnormality. Electronic laryngoscope showed slight hyperemia of the laryngeal mucosa. The findings were in accordance with changes in chronic laryngitis. Brain magnetic resonance imaging (MRI) on the second day after admission showed no obvious abnormalities. **A** Cervical spine MRI on the second day showed multiple cervical disc herniations. **B** Repeat brain MRI on the 4th day showed no obvious abnormality. **C** Head CT showed subarachnoid hemorrhage. **D** Repeat head CT showed that the subarachnoid hemorrhage has been absorbed after one month. **E** Repeat brain MRI on the 55th day showed no abnormality

A 52-year-old man was admitted in November 2020, reporting a tension headache for 20 days. Brain computed tomography (CT, Fig. 1A) showed no obvious abnormality in the patient. He had a history of chronic pharyngitis and had felt pharyngeal discomfort 1 week before admission. An electronic laryngoscope showed chronic laryngitis (Fig. 1B). He had no history of major trauma, toxic exposure, or hereditary disease. Physical examination at admission showed clear consciousness and mild dysarthria but no other abnormal neurological signs.

Laboratory tests showed normal blood tests and blood coagulation parameters. Blood sugar (7.62 mmol/L) and homocysteine (61.82 μmol/L) levels were increased. Blood electrolyte and thyroid function levels were normal. Syphilis and human immunodeficiency virus antibodies were negative. Carcinoembryonic antigen, alpha-fetoprotein, squamous cell carcinoma antigen, carbohydrate antigen (CA) 724, prostate-specific antigen, neuron-specific enolase, alpha-fetoprotein, and cytokeratin 19 fragment levels were all normal. CA 199 (107 U/mL, reference range: 0–37 U/mL) and CA125 (36.9 U/mL, reference range: 0–35 U/mL) levels were increased. A 24-h dynamic electrocardiography showed occasional atrial and ventricular premature beats and ST-T changes. An initial brain magnetic resonance imaging (MRI, Fig. 1C) and brain magnetic resonance angiography showed no obvious abnormalities. Cervical spine MRI (Fig. 1D) showed cervical disc herniation.

On Day 2, the patient had an unsteady gait, distortion of the commissure, and mild panic. Repeated MRIs on Day 2 showed no apparent abnormalities (Fig. 1E). On Day 4, he had developed profuse sweating, dizziness, paroxysmal muscle spasms, and brisk tendon reflexes (Timeline). We initially diagnosed a suspected stroke. We started treatments to improve cerebral circulation, anti-platelets, lower lipids, and to releve his symptoms. However, his symptoms did not improve significantly.

On Day 11, the patient had a subarachnoid hemorrhage (Fig. 1F) caused by a fall, which could have occurred due to a tonic seizure. Physical examination at that time revealed drowsiness and a stiff neck. The patient repeatedly experienced loss of consciousness, clonic seizures, panic attacks unresponsive to olanzapine, and severe anxiety. Paroxysmal inspiratory laryngeal stridor (see Video) without three depression signs, obvious hypoxemia on a hand-held pulse oximeter, and cyanosis of the lips requiring occasional ventilation in status epilepticus were also observed. An electroencephalogram revealed frequent outbreaks of multispike and slow waves on all channels. Multiple myoclonic seizures, a single generalized tonic-clonic seizure, and a single generalized tonic seizure were detected on electromyography. We administered antiepileptic drugs (e.g., continuously pumped sodium valproate, intravenous diazepam, and oral levetiracetam), fluid rehydration, hemostasis, and symptomatic treatment. However, his epilepsy was not well controlled. Repeated head CTs (Fig. 1G) showed that the subarachnoid hemorrhage was absorbed after 1 month, but his condition did not improve.

On day 45, we performed cerebrospinal fluid (CSF) analysis and detected an anti-glycine receptor (anti-GlyR) antibody (1:100). The CSF culture was negative for bacteria, tubercle bacillus, and fungi. Antibodies for demyelination disease (including anti-aquaporin-4, anti-myelin basic protein, anti-myelin oligodendrocyte glycoprotein, anti-glial fibrillary acidic protein), autoimmune encephalitis (including anti-GAD, anti-NMDA, anti-IgLON5, anti-LGI1, anti-CASPR2, anti-DPPX, and anti- GABA), and paraneoplastic syndrome (including anti-Hu, anti-Ri, anti-Ma 1 and 2, anti-CV2, anti-Yo, anti-Tr, and anti-SOX1) in CSF were negative. Electronic gastroscopy was also performed. Electronic colonoscopy and biopsy showed a colon tubular adenoma with low-grade intraepithelial neoplasia.

We suspected anti-GlyR antibody-associated PERM. We began treatment with intravenous methylprednisolone (500 mg/d for 3 days, 240 mg/d for 3 days, 120 mg/d for 3 days, 80 mg/d for 3 days, which was switched to oral prednisone 60 mg/d for 1 month) and intravenous immunoglobulin 22.5 g/d for 5 days. His symptoms improved significantly, and no abnormalities were found on a repeat MRI (Fig. 1H). He was discharged after 13 days of immunotherapy. At 5 weeks after his discharge, the patient reported that the panic attacks, anxiety, and diaphoresis had improved. His gait was normal with no relapse of epilepsy, and the inspiratory laryngeal stridor almost disappeared. By contrasting the symptoms and signs before and after immunotherapy, the neurological symptoms (including dysarthria, dizziness, gait, and epilepsy), nonspecific symptoms (including atypical emotions and autonomic nervous dysfunction), and related signs were significantly improved, most returning to normal. He was satisfied with the treatment received and his recovery.

## Discussion and conclusions

To date, a case of inspiratory laryngeal stridor as a clinical feature of PERM, which may be related to laryngeal spasm, and hypoventilation related to respiratory spasm has not been reported. The disease progresses rapidly, and if not treated in a timely manner, the patient may develop further respiratory failure.

GlyRs are chloride channels that are present mainly in the brainstem and spinal cord. GlyRs are also alkaloid strychnine targets associated with muscle spasms and cramps, muscle stiffness, agitation, stimulation-evoked seizures, myoclonus, respiratory failure, and sometimes death [4]. Antibody-mediated inhibition of the GlyRs on the brainstem nuclei or spinal inhibitory interneurons may cause continuous firing of motor neurons. The excessive and paroxysmal response to various afferent impulses leads to increased stiffness, brainstem signs, trismus, myoclonus, spasms, or hyperekplexia [5]. GlyR-Abs might disrupt glycinergic inhibition mechanisms, causing an excessive startle reflex.

Some patients had a prodrome, such as low mood, hallucinations, involuntary tic-like jerks [2], and flank tingling [6]. These nonspecific prodromal symptoms can last for several weeks to months. Early recognition of prodrome is essential for diagnosis, but it is also difficult to identify.

Our patient had cyanosis, anxiety, brain stem dysfunction, profuse sweating, and palpitations. He also had inspiratory laryngeal stridor, which was followed by a clonic seizure. Similar to that observed in our patient, cyanosis secondary to hypoventilation may be associated with laryngospasm in people with epilepsy. Hypoventilation can be the result of restricted respiration due to high myodynamia (e.g., laryngospasm), but it also may be a manifestation of brain stem dysfunction. Our patient underwent a laryngoscopy for an upper airway obstruction, thought to be related to laryngeal spasm, which was ultimately ruled out. There is a report in the literature that hypoventilation improved with diazepam [7, 8]. Our patient’s symptoms improved with clonazepam. Hypoventilation is a serious complication of anti-GlyR-related PERM, at times manifesting with worsening rigidity and spasms in some patients [7]. We need to pay attention to hypoventilation episodes, especially when exposed to respiratory suppressants, as these episodes increase the risk of respiratory failure.

In anti-GlyR-related PERM, psychological symptoms include phobias [8, 9], irritability, anxiety, and behavioral changes [8]. Generalized anxiety and depression are also frequently reported [6]. We observed hyperekplexia and severe anxiety in our patient, which improved following immunotherapy.

In addition, our patient had facial paralysis, dizziness, dysarthria, and pharyngeal discomfort. Dysphagia [8] and diplopia [2, 8] have also been reported in some patients.

In our patient, multiple myoclonic seizures were detected on electromyography. No painful muscle spasms were noted, and their absence has also been noted in other reports [10]. Rigidity and painful muscle spasms are the core clinical features of PERM [2]. Generalized myoclonic jerks were triggered by sensory stimulation but also occurred spontaneously [7, 8].

Our patient did experience an unexpected generalized tonic seizure, which can frequently lead to falls and fractures. We found similar reports [9] in the literature. Although epilepsy is not the core symptom of PERM, anti-GlyR-related PERM can show intractable epilepsy [9, 10], as in our patient. Immunotherapy shows a beneficial effect.

Finally, our patient had profuse sweating and palpitations. Some patients reported in the literature had high blood pressure and hyperthermia [8]. Patients are often reported to have prominent autonomic symptoms or even autonomic failure [11].

Our patient had normal cranial and spinal MRI scans with gadolinium. This finding is consistent with most MRIs in patients with GlyR-Abs-related PERM [6, 8, 12]. The CSF results in most patients were normal, with a minority of patients having mild inflammation [2, 6]. We found a colon tubular adenoma with low-grade intraepithelial neoplasia and rising CA levels in our patient. Malignancy screening in PERM is important. Approximately 10–20% of patients have an underlying malignant [1] or benign tumor, such as thymoma [3]. However, many patients do not have tumors [7]. We have recommended long-term follow-up for this patient.

Anti-GAD-Abs was not detected in our patient. Since PERM and SPS disorders can have overlapping clinical features, both GlyR- and GAD-Abs should be measured in patients with these clinical characteristics. Serum GlyR-Abs are the primary source of antibodies, and the titers are often higher than those in CSF [2]. However, the clinical disease will depend on the access of antibodies from the serum to the brain [10]. The antibody titers can correlate with disease severity [6]. With immunotherapy, the antibody titers can be decreased [2, 12].

Immunomodulatory pulse therapy with steroids and immunoglobulin resulted in a significant and rapid improvement of symptoms in our patient. Many patients showed substantial and sustained improvement with immunotherapy, particularly combinations of corticosteroids [13], plasma exchange [14], intravenous immunoglobulin, or even immunoadsorption treatment [12]. Patients with GlyR-Abs often improve with aggressive immunotherapy, and a mild decline in GlyR antibody serum titers indicates clinical improvement [10]. There is often a clear response to immunotherapy in patients with neurological disorders associated with GlyR-Abs [11, 15]. For some patients with severe PERM, long-term immunotherapy is needed [2, 7] Relapses are not rare [6, 8], and subsequent immunotherapy treatments are effective [2, 6]. For some patients with refractory drug-resistant epilepsy, active immunotherapy is conducive to control epilepsy rapidly. Patients may not need long-term use of antiepileptic drugs after PERM is controlled. PERM typically progresses and then stabilizes over weeks to years. Some patients may relapse, and these episodes may lead to some sequelae [2]. The prognosis is variable, but early, aggressive immunotherapy may contribute to a better prognosis. We have summarized the clinical features of the PERM patients with only positive GlyR-Abs according to the reported cases (see Table 1).

**Table 1:** Clinical features of patients with PERM with only positive glycine receptor antibodies

Authors	Age/Sex	Clinical Features	Brain/spinal MRI	CSF analysis	Immunotherapy	Outcome
Stern W M [2].	40/Male	Dyspnea, ophthalmoplegia, severe limb rigidity, stimulus-sensitive myoclonus, profuse sweating, and episodes of tachycardia. After 7 months, he experienced a relapse	Normal/—	Mildly inflammatory	IVIG, PLEX, oral corticosteroids	At 6 months, the primary symptoms had improved.
Hutchinson M [6].	54/Male	Two weeks of worsening with brief frequent violent jerks, followed by bilateral ptosis, partial horizontal gaze palsies, and rigidity	Normal/normal	Mildly inflammatory	Corticosteroids, PLEX, IVIG, Cyclophosphamide	Mild spinal rigidity, walks 200 m with one stick, and works part time
Bourke D [7].	55/Male	Stimulus-induced hyperekplexia and rigidity in the lower limbs and trunk	Normal/normal	Normal	Methylprednisolone pulse therapy and IV IG	A gradual reduction in the frequency and severity of hyperekplexia
Bourke D [7].	58/Female	Stiffness in the legs and body jolts associated with hypoventilation at times leading to loss of consciousness	Normal/ normal	Normal	Without immunotherapy, except for diazepam and clonazepam	Symptom improvement
Mas N [8].	48/Male	Paresthesia, irritability, dysgeusia, and severe diurnal hypersomnia, followed by progressive rigidity, trismus, leg spasms, facial flushing, and diaphoresis.	Normal/ normal	–	Oral corticosteroids, IVIG	Only leg stiffness was partially improved.
Mas N [8].	33/Female	Diplopia, dysphagia, and gait ataxia, followed by rigidity of lower extremities with painful spasms, involuntary jerks, and contracture of ankles and urinary retention. Relapse of illness 22 months later.	Normal/ normal	Normal	Corticosteroids, IVIG	A progressive complete recovery
Mas N [8].	60/Male	Progressive dysphagia, followed by rigidity of his legs with painful spasms, diplopia, facial numbness, and severe dysautonomia	Normal/—	Normal	–	A persistent vegetative state and ventilator dependent
Schmidt C [9].	21/Male	Generalized pruritus, paroxysmal fear, and disturbance of sleep, followed by progressive gait ataxia, postural instability, and generalized myoclonic jerks	Normal/normal	Normal	Methylprednisolone pulse therapy	Substantial clinical improvement
Wuerfel E [10].	4/Male	Drug-resistant focal epilepsy, temper tantrums, headache, clumsiness, and intermittently impaired speech	Normal/—	Normal	Methylprednisolone pulse therapy.	Expeditious improvement of within 8 weeks. No epileptic seizures
Kenda J [12].	67/Male	Speech and swallowing difficulties, leg weakness, shortness of breath, twitching of his face and limb muscles, and respiratory failure. The disease relapsed 1 year later.	Normal/—	Unremarkable	Methylprednisolone, IVIG, PLEX, immunoadsorption, azathioprine	Improvement of rigidity, hyperekplexia, and ophthalmoparesis over the following months.
Borellini L. [13]	60/Female	Low back pain and progressive rigidity of the trunk and lower limbs, followed by pruritus, dysphonia, hyperhydrosis, and urinary retention	Normal/normal	Normal	Corticosteroids PLEX	At the 1-year follow-up, the neurological findings were normal.
Peeters E [14].	37/Male	Muscle jerks, painful spasms, falls, supranuclear upward gaze palsy and slow saccades, dysphagia, constipation, urinary retention, and paresthesia.	Normal/ normal	–	Methylprednisolone pulse therapy, followed by oral therapy; PLEX	Mild hypertonia and slowed upward saccades persisted at discharge.

*CSF* Cerebrospinal fluid, *GlyR* Glycine receptor, *IVIG* Intravenous immunoglobulin, *MRI* Magnetic resonance imaging, *PLEX* Plasma exchange, *PERM* Progressive encephalomyelitis with rigidity and myoclonus; —, no mention

A limitation of this report is that the follow-up duration was short, and we did not perform PET-CT. A large-sample, multi-institution research program is needed in this respect.

In conclusion, our case highlights that inspiratory laryngeal stridor is one of the important symptoms of PERM. Patients with this symptom may further develop respiratory failure. It is vital to identify the clinical characteristics of PERM early to allow early aggressive immunotherapy, which may influence the prognosis. This case report broadens the clinical presentation spectrum of anti-GlyR-Abs and emphasizes the need to prevent serious complications early.

## Supplementary Information

**Additional file 1.** The video was recorded during the patient’s hospitalization. The patient had a sudden inspiratory laryngeal stridor and felt dyspneic, followed by clonic seizures, and then cyanosis of the lips within a a few minutes.

**Additional file 2.**

## Data Availability

Not applicable.

## Abbreviations

CA: Carbohydrate antigen
CT: Computed tomography
CSF: Cerebrospinal fluid
GlyR-Abs: Glycine receptor antibodies
MRI: Magnetic resonance imaging
PERM: Progressive encephalomyelitis with rigidity and myoclonus.
SPS: Stiff-person syndrome
