# Supplementary material for: Inspiratory laryngeal stridor as the main feature of progressive encephalomyelitis with rigidity and myoclonus: a case report and literature review
Source: BMC Neurol. 2022 Jan 28;22:42. doi: 10.1186/s12883-022-02555-y (PMC8796497; doi:10.1186/s12883-022-02555-y)
Supplement: Supplementary file 2 — Additional file 2. [file 12883_2022_2555_MOESM2_ESM.doc]

**Figure 1:** Case Timeline

SAH was absorbed, no clinical improvement

Tension headache for 20 days

Traumatic SAH

by unconsciousness, clonic

seizure, phobias,anxiety, paroxysmal inspiratory

laryngeal stridor

Substantial clinical improvement

by unconsciousness, clonic

seizure, phobias,anxiety, paroxysmal inspiratory

laryngeal stridor

On admission

Day 4

Day 11

Month 1 after SAH

Day 45

Day 2

5 weeks after discharge

Anti-GlyR-Absdetected, IVIG and IV steroids

Panic, diaphoresis, dizziness, muscle spasms

Unsteady walk, dysarthria, panic

Unconsciousness, seizure, inspiratory

 laryngeal stridor

SAH: sub-arachnoid hemorrhage; GlyR-Abs: Glycine receptor antibodies; IVIG: intravenous immunoglobulin
